# The role of educational technology in medical education

**Published:** 2014-10

**Authors:** ZAHRA SAFFARI, FARNAZ TAKMIL, RAHMATALAH ARABZADEH

**Affiliations:** 1 Quality Improvement in Clinical Education Research Center, Shiraz University of Medical Sciences, Shiraz, Iran; 2Shiraz University of Medical Sciences, Shiraz, Iran

## Dear Editor

Being one of the most effective tools for educational system improvement, educational Technology plays an important role in learning facilitation. In order to have a deeper, more effective and long lasting learning impact, this systematic approach designs, implements and evaluates the teaching- learning process, using specific purposes, new methods of psychology and communication sciences and also human and non-human resources (1).

A fruitful and effective educational system which results in actual learning improvement cannot be achieved unless its faculty members become competent. To achieve this goal, not only they must attain and/or maintain academic qualifications, especially in their teaching area, but also be familiar with the newest communication and teaching methods and equipped with educational and professional skills. 

Considering the growing movement of education towards the new technologies and the Ministry of Health and Medical Education tendency for upgrading the educational technology and virtual learning, the need for experts in education technology was clear. Therefore, given its mission which focuses on scientific promotion and academic training improvement, in an cooperation with shiraz educational development center along with Center of Excellence for electronic learning`s staff and faculty members, Shiraz Educational Technology unit, established the Master of education technology courses (2).

Education’s technology and E-Learning, have arises a condition in which many educational goals, such as independent learning, self-directed learning, learning regardless of time or place, collaborative learning and providing immediate feedbacks and assessment of learning, appears more achievable.

Electronic medical education has become very popular in developed countries and is rapidly developing, since it has educational value and the tremendous broadening audience through educational programs.

Considering the fact that currently, while the faculty members have to learn most of the new means of teaching, for most of their students, these new means of education such as computers and other associated applications are not really consider technology since they are not only completely familiar with but also very capable of utilizing them. Therefore, while faculties are trying to learn these new methods of transferring information, students’ expectations are getting higher each day, Hence it seems that educational technology mastery is an important neglected competency in faculties which mandates an especial training program for the universities’ upgrading and improvement. 
